# High Intensity Interval Training (HIIT) Improves Cardiorespiratory Fitness (CRF) in Healthy, Overweight and Obese Adolescents: A Systematic Review and Meta-Analysis of Controlled Studies

**DOI:** 10.3390/ijerph17082955

**Published:** 2020-04-24

**Authors:** Rhona Martin-Smith, Ashley Cox, Duncan S. Buchan, Julien S. Baker, Fergal Grace, Nicholas Sculthorpe

**Affiliations:** 1 Movement Behaviours, Health and Wellbeing Research Group, Department of Sport and Physical Activity, Edge Hill University, Ormskirk, Lancashire L39 4QP, UK; 23730170@edgehill.ac.uk; 2 Institute of Clinical Exercise and Health Science, University of the West of Scotland Lanarkshire Campus Lanarkshire, Scotland G72 0LH, UK; duncan.buchan@uws.ac.uk (D.S.B.); nicholas.sculthorpe@uws.ac.uk (N.S.); 3 Centre for Health and Exercise Science Research, Department of Sport, Physical Education and Health, Hong Kong Baptist University, Kowloon Tong, Hong Kong, China; jsbaker@hkbu.edu.hk; 4 School of Health & Life Sciences, Federation University, Mt Helen, Ballarat, VIC 3350, Australia; F.Grace@federation.edu.au

**Keywords:** high intensity interval training, cardiorespiratory fitness, adolescents

## Abstract

*Background*: High Intensity Interval Training (HIIT) is a sustainable and effective method for improving Cardiorespiratory Fitness (CRF) in adolescents. HIIT is proven to produce equal or greater improvements in CRF when compared to moderate intensity continuous exercise (MICE) in adolescents. *Methods*: The studies included were considered eligible if: (1) Participants were adolescents (11–18 years old); (2) Examined changes in CRF measured either directly or indirectly; (3) Included a non-exercising control group or MICE comparison group; (4) Participants were matched at enrolment; (5) Reported HIIT protocol information; (6) Provided HIIT intensity. A meta-analysis was conducted to determine the effect of HIIT on CRF. Meta-regression and moderator analyses were performed out to quantitatively examine moderators of protocol design on CRF improvements. *Results*: HIIT displays a moderate effect to improve CRF (*g* = 0.86, 95% CI 0.518–1.106, *p* < 0.001). Neither study duration (weeks), nor total or weekly accumulated HIIT volume (min) displayed any significant moderation effect on pooled improvement on CRF (*p* > 0.05). *Conclusions*: HIIT is an effective method to improve CRF in adolescents, irrespective of body composition. Notably, meta regression analysis identified that prolonged high volume HIIT programs are similarly effective to short term low volume HIIT programs. This becomes of particular interest for those involved in school curricula, where short HIIT exercise may provide a pragmatic adjunct to the health benefits of Physical Education (PE) lessons.

## 1. Introduction

Despite the associated health benefits of physical activity (PA), many children and adolescents do not currently achieve the proposed PA recommendations of 60 min of moderate to vigorous physical activity (MVPA) per day [1,2]. This is further compounded by approximate 65% decrease in PA during adolescence [3,4,5,6]. It is established that adolescents are more physically active within the school environment compared to evenings and weekends [7], with availability/lack of time being cited as an obstacle to achieving daily PA recommendations [8]. Additionally, the school environment provides access to PA independent of background or socioeconomic status [9]. It has been suggested that the school environment may provide a safe and accessible environment that allows for PA participation independent of socioeconomic status [9,10].

Cardiorespiratory fitness (CRF) has been identified as a strong predictor of cardiovascular (CV) and cardiometabolic disease outcomes in adolescents [11]. Given the health risks associated with poor CRF and the risk of these continuing into adulthood [12], efficient methods are required for improving and maintaining CRF during childhood and adolescence, and this is an ever-important yet under-represented public health concern. However, many school-based interventions have been proven unsuccessful in improving PA, CRF and health outcomes in children and adolescents [9,13,14,15]. A recent meta-analysis of cluster randomised controlled trials on school-based PA interventions reported no effectiveness at increasing children’s accelerometer measured daily time spent on MVPA [9], thus suggesting that alternative school-based interventions are required to increase PA, CRF and health outcomes in children and adolescents.

High intensity interval training (HIIT) interventions, have emerged in recent years as a time-efficient means to improve CRF in as little as two weeks in adults [16,17,18,19,20]. Moreover, HIIT when compared to traditional endurance training or moderate intensity continuous exercise (MICE) can produce similar and even superior changes in physiological and physical performance and health related outcomes, but with a sustainably reduced exercise duration and volume [17,21]. Recent studies have also demonstrated the potential for running based HIIT to improve CRF or maintain healthy levels of CRF alongside Physical Education (PE) activities within the school environment [22,23,24,25,26,27,28]. Nonetheless, it remains unclear as to the quantity and intensity of HIIT required to achieve CRF improvements and whether HIIT is more effective in obese adolescents when compared to healthy adolescents.

Recent systematic reviews and meta-analyses of adolescent HIIT reviewed the effects of HIIT on CRF in comparison to control groups in normal weight, overweight and obese adolescents [26,29,30,31]. However, there were many limitations of these systematic reviews and meta-analyses including: (1) a number of key studies were excluded including the first HIIT study on adolescents; (2) only studies that presented CRF values in tables or within text or studies that included direct measures of CRF were included (no indirect studies) which may limit the upscaling of HIIT interventions in an applied setting; (3) limited comparator analysis to both the effects of HIIT vs. no exercise and HIIT vs. MICE as well as the inclusion of uncontrolled groups; (4) limited comparator analysis to both the effects of HIIT vs. no exercise and HIIT vs. MICE and including uncontrolled studies (studies using a HIIT group with no MICE or NO exercise comparison group [31]. Furthermore, recent systematic reviews on the effects of adolescent HIIT on CRF call for further research to identify the difference in inactive (unfit), overweight and obese children and adolescents as these reviews suggest that overweight and obese children and adolescents are more likely to benefit from HIIT [26,31]. However, there is currently a lack of quantitative examination to support or reject this hypothesis. Another key variable in adolescent HIIT research is the volume of HIIT exercise per session and the duration (number of sessions, minutes per week and number of weeks) required to induce an improved CRF. Nonetheless, it remains unclear which duration (weeks) and (minutes per HIIT session, minutes per week and minutes per intervention duration) of HIIT is required to induce improvements in CRF in adolescents.

Therefore, the objectives of this systematic review and meta-analysis were: (1) To expand the search parameters to include and compare both indirect calorimetry (laboratory) and predicted (field) measures of CRF, thus exploring how different methods of CRF measure may influence research outcome, and to provide a consensus on the most appropriate method to conduct future research. (2) To use moderator analysis to compare the effects of HIIT vs. either MICE or no Exercise on CRF. (3) To use moderator analysis to compare the effects of HIIT on CRF in unfit vs. fit adolescents and obese vs. healthy adolescents. Finally, to use meta-regression analysis to determine the amount of HIIT in terms of total minutes, number of sessions per week and total length required to induce improvements in CRF in adolescents.

Finally, addressing adolescent HIIT data reported in both direct and indirect measures of CRF will provide more data to enhance our understanding of the efficacy of HIIT training in adolescents. Furthermore, the pooling together of these data builds upon previous analyses when providing inference on the impact of weight status, HIIT protocol and baseline fitness levels on future research design and HIIT prescription. The aim of this systematic review and meta-analysis was to primarily investigate if a statistically significant difference existed in the effect size of HIIT on CRF when comparing studies that used: indirect calorimetry (laboratory) vs. predicted (field) measures of CRF, MICE or No Exercise as the control group, and unfit vs. fit or obese vs. healthy weight adolescents A secondary aim of this systematic review and meta-analysis was to determine if there was a dose response relationship between total HIIT min week^−1^ or total program length and improved CRF in adolescents.

## 2. Materials and Methods

### 2.1. Protocol and Search Procedures

**Figure 1:**
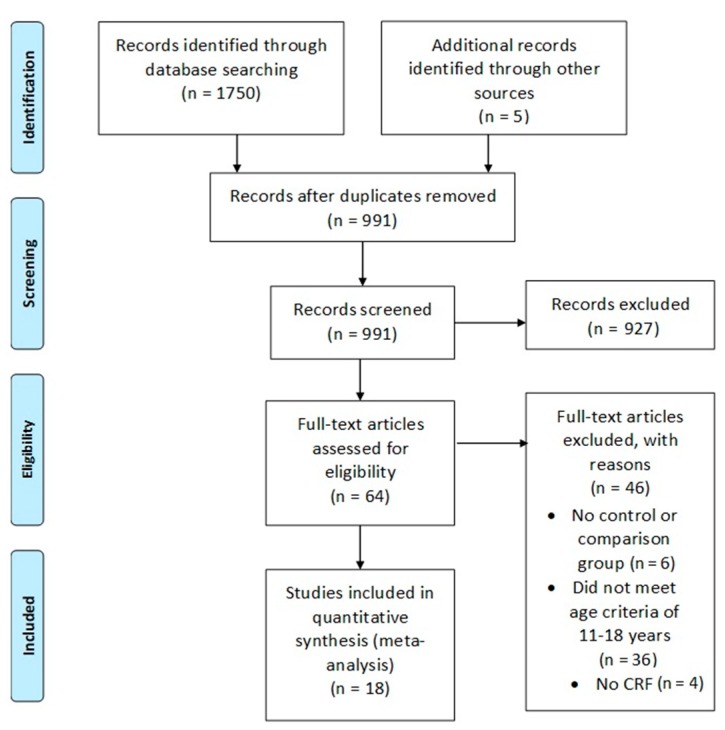
Preferred Reporting Items for Systematic Reviews and Meta-Analyses (PRISMA) flow diagram to show each stage of the systematic eligibility process. Note: CRF = Cardiorespiratory Fitness.

This systematic review and meta-analysis were carried out following the Preferred Reporting Items for Systematic Reviews and Meta-Analyses (PRISMA) Statement [32] and procedures are reported in Figure 1. Electronic database searching was carried out using PubMed, Web of Science, SPORT Discuss and MEDLINE using all available records up to January 2019. The following search term strings were used: “High intensity interval training OR intermittent training OR High intensity interval running OR Aerobic interval training OR Sprint interval training AND adolescents”. The literature search and data extraction were performed independently by two authors (R.M.-S. and F.G.). Any disagreements were resolved in a meeting with R.M.S., D.S.B., A.C., J.S.B., N.S. and F.G. Studies that had no clear relevance or were review/meta-analysis articles were removed from the database before the assessment of all other articles using our inclusion criteria. The results were further limited to full text and English language with abstracts and duplications removed. Reference lists from retrieved full text articles were also examined for any other potential studies.

### 2.2. Inclusion Criteria

The meta-analysis included only full text randomised control trials (RCT) or control trials (CT) with cohort studies being excluded. An inclusion criterion was set as follows: (1) An adolescent population within the age range of 11–17 years. (2) Included either a non-exercising (CON) or moderate intensity exercising comparison group (MICE). (3) Measured CRF either by direct or indirect methods. (4) Reported intensity of HIIT (% of maximum heart rate (% HRmax) or maximum aerobic speed (MAS) or % of maximal oxygen uptake (% V̇O_2max_) or % ventrally threshold (% VT)). (5) HIIT intervention ≥2 weeks in length. (6) Drop out <20%.

### 2.3. Data Extraction

Data extraction was performed by one author (R.M.-S) which allowed for the extraction of study characteristics including author, year, number of subjects, subject age range or mean, study duration, work: rest ratio, frequency of training, total HIIT minutes per session and per week, mean and standard deviation PRE and POST training, V̇O_2max_ in mL/kg/min and effect size. If study characteristics were not presented in the text, figures or tables, the corresponding authors were contacted in order to retrieve study characteristics. Similarly, where V̇O_2max_ was not presented in tables, data was extracted from figures using calibrated figure measures (ImageJ software tool, ImageJ version 1.46r, National institutes of Health, Bethesda, Maryland, MD, USA).

To assess the risk of bias in the 18 selected studies, two reviewers (R.M.-S and F.G) independently assessed the studies (Table 1). For the 18 studies included an 8 item checklist was created using the Cochran tool for assessing risk of bias in randomised trials [33]. The check list included the following eight items: (A) Participants were randomly assigned to groups. (B) The groups were similar at baseline. (C) Blinding of assessors taking primary outcomes. (D) Adequately powered groups. (E) Included a non-exercising or moderate intensity comparison group. (F) HIIT intensity was included. (G) HIIT total time included. (H) Group x Time interaction and effect sizes included. If the study included these items a “✓” was given under the item; if the study did not include the item a “X” was given under the item. If it was unknown whether the study included the item a “NA” was given under the item. From this analysis each study was awarded an overall risk of bias score between 1–8.

The PubMed search and the author’s search of own bibliography library identified a total of 1750 studies. The 1750 identified studies were further limited to full text and abstracts which narrowed the total down to 991. From this, 927 were excluded and a total of 64 studies were retrieved for detailed assessment for eligibility for inclusion. A further 46 studies did not meet inclusion criteria. Reasons for exclusion included: (A) No control or comparison group, *n* = 6. (B) Did not meet age criteria, *n* = 36. (C) Did not measure CRF, *n* = 4. Hence, a total of 18 studies met inclusion criteria and were included in the meta-analysis (Figure 1).

### 2.4. Publication Bias

**Figure 2:**
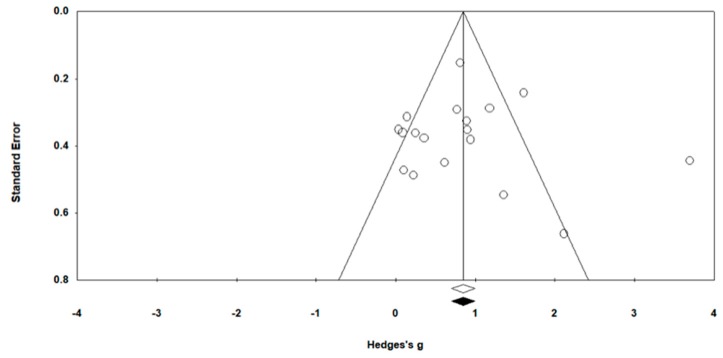
Funnel Plot of Standard Error by Hedges’ g.

Publication bias was assessed using Egger’s statistic test, where bias was deemed to be present at *p* ≤ 0.05 [46]. Corresponding funnel plots were created for visual interpretation, followed by an Egger’s statistic to confirm or refute publication bias (Figure 2). Egger’s analyses suggest that publication bias was not present (*p* > 0.05) finding.

### 2.5. Risk of Bias

Risk of bias scores are provided in Table 1. A total of 16/19 studies reported low-moderate risk of bias, 2 studies reported moderate risk of bias and 0 studies reported high risk of bias (Table 1). Participants randomly allocated to groups occurred in 6/18 studies [36,37,40,41,44,45] (Table 1). All 18 studies reported similarity between groups at baseline (Table 1). Blinding of all assessors for primary outcomes was present in 3/19 studies [34,38,39] (Table 1). Out of 18 studies, 5 studies did not report calculating power for group size [22,36,38,39,40] (Table 1). All studies included either a MICE group (*n* = 14) [22,23,25,27,28,36,38,39,42,43,45] or both a non-exercising control group (CON) and a MICE group (*n* = 4) [24,35,40,41] (Table 1). However, one study provided no information on the control group’s activity [34]. All 18 studies reported intensity of HIIT intervention, and 11 studies reported effect sizes (Table 1).

### 2.6. Data Synthesis and Analysis

Random effects Meta-Analysis was carried out to determine the pooled effect size of HIIT on CRF using Comprehensive Meta-Analysis software (Comprehensive Meta-Analysis Software Version 2.2.064, Englewood, NJ, USA). A random effects model was considered more appropriate for this review to account for the expected heterogeneity between measures. Pooled weighted standard deviations were used as per the Hedge’s g formula and based on a positive effect direction for all interventions [47]. Separate moderator analysis were conducted to determine if there were statistically significant differences in the pooled effect sizes of HIIT on CRF: (i) in studies using either predicted or direct measures of CRF, (ii) in studies which used a MICE control group versus studies which used a No Exercise control group, and (iii) in studies using fit adolescents versus studies using unfit adolescents. A further examination of studies using obese participants versus studies using healthy weight participants resulted in identical groupings as (iii). Hedges’ g was interpreted using Cohen’s effect sizes, as small (0.2), medium (0.5) and large (0.8) [47]. The precision of the pooled effect size was reported as 95% confidence intervals (95% CI). Overall heterogeneity was determined using Cochrane guidelines: an I^2^; 0% to 40% represents low heterogeneity; 30% to 60% may represent moderate heterogeneity; 50% to 75% may represent substantial heterogeneity; 75% to 100% is regarded as high heterogeneity [33].

## 3. Results

Descriptive characteristics of HIIT studies included in the analysis, in terms of participant characteristics, study design, HIIT protocol type, HIIT volume, length and intensity are reported in Table 2. A total of 7/18 studies used randomised control trial (RCT) [34,35,36,40,41,44,45] with the remaining 11 studies using control trials (CT) [22,23,25,27,28,37,39,42,43]. Participants’ ages ranged between 10.7 and 17 years. Samples were separated into HIIT, CON and MICE groups. A full breakdown of how sample sizes were extracted is provided in Table 2. Sprint running was the favored method of HIIT in 13/18 studies (Table 2). Cycling was used in 3 studies [35,39,44], dance in one [34] and skiing in another [42] (Table 2). Total HIIT length was reported in all 18 studies and ranged between 4 weeks [37,43] and 15 weeks [35] (Table 2). A total of 16/18 studies reported total HIIT duration which ranged between 1 hour to 6 h 20 min (Table 2). Two studies failed to report total HIIT duration [38,42]. All studies (*n* = 18) reported intensity of HIIT intervention in terms of % HRmax (*n* = 11) [23,24,25,34,39,42,43,45] with an average of 89.2% HRmax, 100% max Velocity Threshold (VT) (*n* = 1) [35], 90-95% age predicated maximal heart rate (APMHR) (*n* = 1) [44], 100% V̇O_2max_ (*n* = 1) [38] and 100% MAS (*n* = 4) [22,36,40,41] (Table 2).

Descriptive CRF values are presented in Table 3 and report pre- and post-CRF values from the HIIT intervention, % improvements in CRF and effect size (*g*) as well as statistical significance (*p*). The average HIIT CRF value in the HIIT groups at pre- was 41.1 ± 12.1 mL kg^−1^ min^−1^ and at post- was 44.3 ± mL kg^−1^ min^−1^. In the control groups the average CRF value at pre- was 41.4 ± 12.8 mL kg^−1^ min^−1^ and at post- was 41.7 ± 12.5 mL kg^−1^ min^−1^. The average percentage increase in CRF pre- to post- in HIIT groups was 7 ± 3.7%.

### 3.1. Cardiorespiratory Fitness (CRF) Meta-Analyses

**Figure 3:**
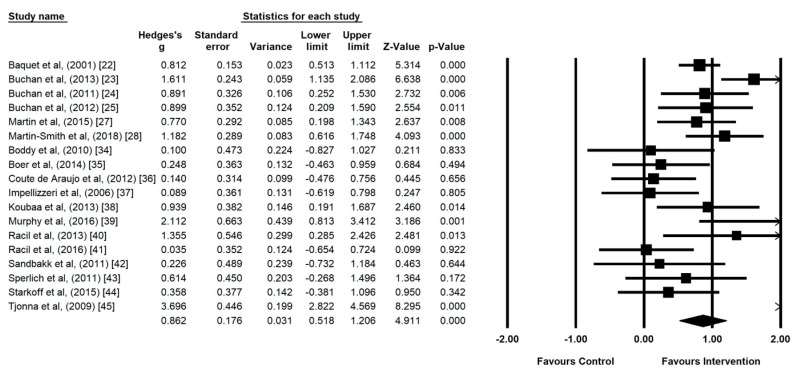
Forest plot of high intensity interval training (HIIT) versus control using random effects model.

All 18 studies were included in the meta-analysis investigating the impact of HIIT on CRF in adolescents (Figure 3) and CRF values are presented in Table 3. The meta-analysis reported moderate heterogeneity between studies reviewed (I^2^ = 78.87, *p* ≤ 0.00). Intervention effect size demonstrated a statistically significant medium effect (*g* = 0.86, 95% CI 0.52 to 1.11, *p* ≤ 0.00).

### 3.2. Body Composition

**Figure 4:**
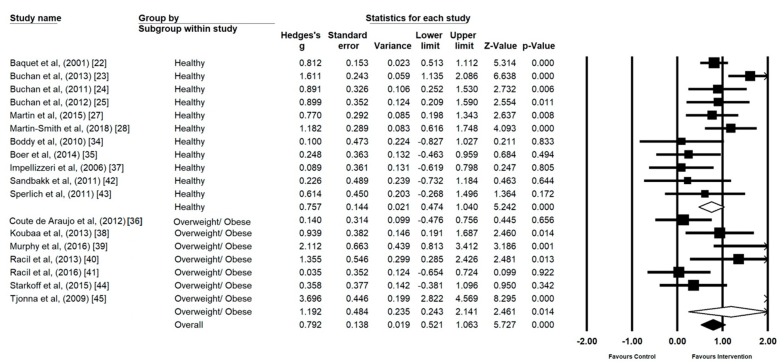
Forest Plot of the Comparison of effect size of high intensity interval training (HIIT) and sprint interval training (SIT) on cardiorespiratory fitness (CRF) in studies using healthy and overweight/obese participants.

CRF interventions demonstrated a large effect on those with a healthy body composition (*n* = 11) (*g* = 0.75, 95% CI 0.47 to 1.04, *p* ≤ 0.00, I^2^ = 56.29) **(**Figure 4). CRF interventions in the overweight and/or obese (*n* = 7) elicited a larger effect, but the results were less homogenous (*g* = 1.19, 95% CI 0.24 to 2.14, *p* = 0.01, I^2^ = 89.54).

### 3.3. Direct vs. Indirect

**Figure 5:**
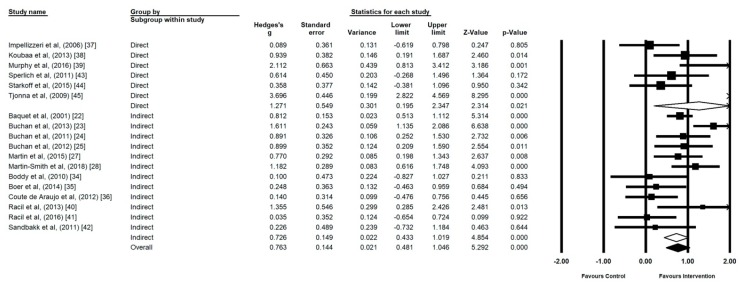
Forest Plot of the Comparison of effect size of high intensity interval training (HIIT) and sprint interval training (SIT) on cardiorespiratory fitness (CRF) of studies assessing CRF via direct measurement of expired gases or indirect prediction.

Direct measures of investigating CRF (*n* = 6) reported large effects with high heterogeneity (*g* = 1.27, 95% CI 0.19 to 2.34, *p* ≤ 0.02, I^2^ = 89.85) (Figure 5). CRF intervention measurements using indirect methods (*n* = 12) were more homogenous and reported smaller effects (*g* = 0.72, 95% CI 0.43 to 1.01, *p* ≤ 0.00, I^2^ = 62.27).

### 3.4. Low Cardiorespiratory Fitness (CRF) vs. High Cardiorespiratory Fitness (CRF)

**Figure 6:**
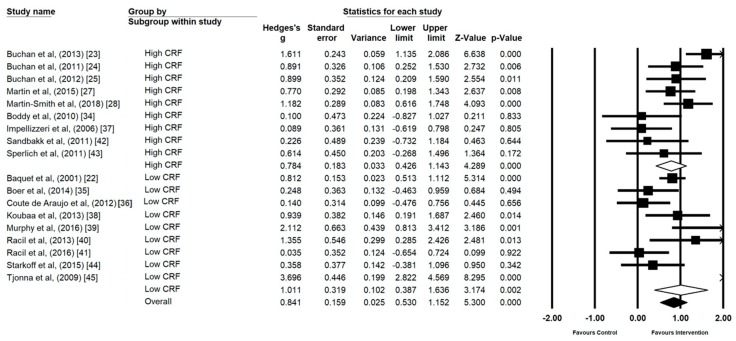
Forrest Plot of the Comparison of effect size of high intensity interval training (HIIT) and sprint interval training (SIT) on cardiorespiratory fitness (CRF) in studies using CRF levels at baseline.

Participants starting interventions with measures of high CRF (*n* = 9) demonstrated a large effect (*g* = 0.78, 95% CI 0.42 to 1.14, *p* ≤ 0.00) (Figure 6). Participants starting interventions with measures of low CRF (*n* = 9) demonstrated a large effect, with statistically significant heterogeneity (*g* = 1.01, 95% CI 0.38 to 1.63, *p* ≤ 0.00, I^2^ = 86.70).

### 3.5. Control Type

**Figure 7:**
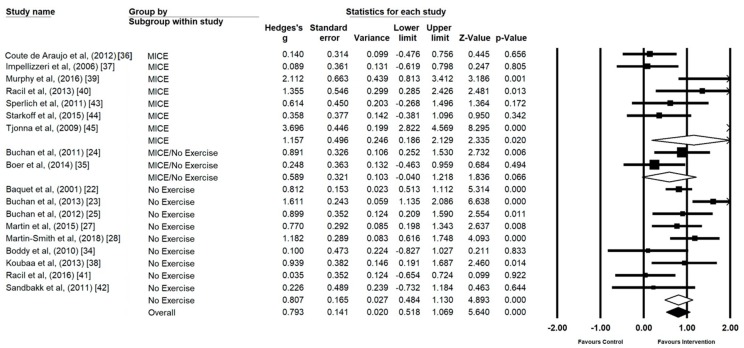
Forest Plot of control group type, individual and group analysis.

MICE as a control type (*n* = 7) demonstrated a statistically significant large effect (*g* = 1.15, 95% CI 0.18 to 2.12, *p* = 0.02, I^2^ = 89.42) (Figure 7). No exercise control groups (*n* = 9) also demonstrated a statistically significant intervention effect (*g* = 0.80, 95% CI 0.48 to 1.13, *p* ≤ 0.00, I^2^ = 61.56). Mixed control groups (MICE and no exercise) were omitted from the analysis as the number was insufficient (*n* = 2). However, mixed control groups are represented within Figure 7 to provide a visual representation.

### 3.6. Moderator Analyses

**Figure 8:**
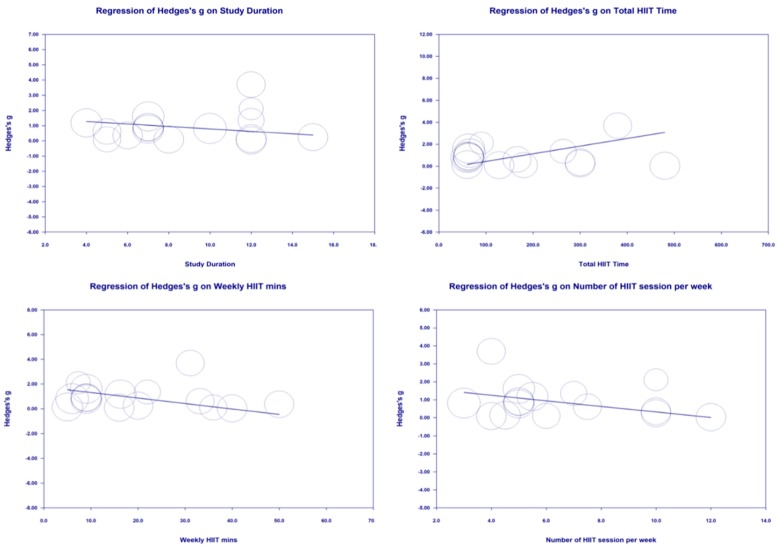
Moderator Analysis of the effects of study duration, total HIIT time, Weekly HIIT minutes (min) and Number of HIIT session per week on CRF.

Study duration (weeks), total HIIT time (min), Weekly HIIT time (min) and number of sessions per week were not significant moderators of the effects of HIIT on CRF in adolescents (*p* > 0.05) (Figure 8).

## 4. Discussion

The main findings of the present study are that HIIT significantly improves CRF compared to non-exercising control or MICE control groups. Furthermore, our moderator analysis indicated that the effect of HIIT is greater in studies that employ a non-exercising control group, whereas studies that compare HIIT to MICE demonstrate a lesser effect size, although still favor the effect of HIIT over MICE training. In addition, moderator analysis demonstrated that there was no difference in the combined effect size in studies that utilised direct measures of $\dot{V}O_{2\mathrm{Max}}$ versus studies that used indirect assessment. In addition, there is no difference in the effect of HIIT when comparing studies that used highly fit adolescents versus those that used adolescents in the lowest quartile. As those in the lower quartile for CRF tended to have higher BMI, this also meant there was no difference when comparing the effect of HIIT in studies using overweight or obese participants with those using adolescents in a healthy weight bracket. Interestingly, the results of the meta-regression indicated that there is no effect of any dose-response relationship between HIIT and either the number of minutes of HIIT performed per week, nor the total length of the HIIT intervention (weeks).

### 4.1. Direct versus Indirect Measures of $\dot{V}O_{2Max}$

In evaluating the ability of a HIIT program to improve CRF, some studies have opted for the more accurate but more time-consuming method of indirect calorimetry with the consequent reduction in participant numbers [35,37,38,40,41,42,43]. Conversely others have used maximal field tests that allow the prediction of maximal aerobic capacity through established equations [22,23,25,27,28,36]. These field tests have the advantage of reduced financial and time costs but are generally regarded as less precise. The different choices of test can influence subsequent meta-analyses, for example, Costigan et al. (2015) [29] included only those studies that used the gold-standard method of indirect calorimetry and consequently were unable to include the largest available study of school based HIIT (using 550 adolescents) [22]. Clearly there are concessions to be made between maximizing the accuracy of CRF measurement and the greater statistical precision of the effect size that comes from higher participant numbers. Furthermore, establishing the usefulness of HIIT to improve CRF in students will require future larger scale, multi-centre evaluations which will most likely require field assessment of changes in CRF. Consequently, before such studies are implemented, it is imperative to establish the equivalence of effect when assessing CRF directly through analysis of expired gases, or through prediction equations following a functional test. The data presented here demonstrate that studies using the two methods do indeed provide equivalence with no difference in the overall effect size between either type of study. Correspondingly, the overall meta-analysis, subsequent moderator analyses, and the meta-regressions reported in the present study all benefit from including twice as many data points (studies) as previous analyses. Furthermore, it confirms the suitability of indirect measures of CRF that may be used in future large scale RCT trials.

### 4.2. Effect of Control Group Type

The present data indicate that the effect size of HIIT interventions are significantly influenced by the type of control group used as a comparator. Costigan et al. [29] reported no difference in the effectiveness of HIIT when comparing studies using non-exercising versus moderate intensity exercise control groups. Such a finding appears at odds with established tenets of exercise physiology, not least as it also suggests that MICE and no-exercise are comparable methods of improving CRF. However, there were some limitations to the analysis by Costigan et al. [29]. Primarily, their analysis only included studies that reported direct measures of $\dot{V}O_{2\max}$ utilising indirect calorimetry which limited the number of included studies to a total of 7. In contrast, by establishing that the effects of HIIT are equivalent regardless of whether $\dot{V}O_{2\mathrm{Max}}$ was measured directly or indirectly, the current study was able to expand the number of studies included in the meta-analysis. As such, this in turn allowed for more meaningful moderator analysis with seven and eight studies included in the MICE and no-exercise groups respectively. Consequently, the present study is a strong indicator that the type of control group that is used as a comparator for HIIT studies has a significant bearing on the resultant effect, a conclusion that is more physiologically plausible. Correspondingly, and in line with similar discussion in the medical literature [48], this study suggests that future studies should compare HIIT to ‘usual treatment’ (in reality this will frequently be ‘MICE’) rather than to non-exercising controls.

### 4.3. Utility in High Fit and Low Fit Groups

Given that $\dot{V}O_{2\max}$ is finite, it was anticipated there would be diminishing returns from HIIT training when increasing aerobic fitness of participants between studies. Indeed, some recent commentators have suggested that HIIT is most appropriately employed in adolescents with low levels of maximal aerobic capacity [31]. This hypothesis was tested by using pre-intervention data to classify each study according to their participant’s aerobic capacity (Figure 6). The result, that participants in the highest quartile of aerobic fitness enjoyed similar increases in $\dot{V}O_{2\max}$ as those in the lowest quartile, was unexpected. The reasons for the equivalence of effect may be due to several factors. It is possible that the general decline in aerobic fitness of UK adolescents of 0.36% per year [49] means that there is greater scope for improvement than anticipated even within those in the top quartile. Similarly, recent data indicates that school PE in the absence of specific training results in a fall in aerobic capacity in the weeks following return to school after summer [27]. The timing of these studies using fit adolescents i.e., first term of school following a summer vacation period, could also be a factor. It may also be worth noting that, as $\dot{V}O_{2\max}$ is routinely reported relative to body mass, splitting the studies into those with obese/overweight participants, versus those with healthy weight participants, resulted in almost identical groupings. As a result, moderator analysis examining the effect of HIIT in studies using healthy weight adolescents versus overweight/obese adolescents also resulted in no statistically significant difference. Consequently, despite the plausibility of participants with low fitness, or high BMI benefiting most, the present data indicates that HIIT is equally beneficial across a broad range of maximal aerobic capacities and BMI categories.

### 4.4. Training Duration

The lack of any dose response between CRF and the duration of HIIT interventions is another unexpected finding of this study. In order to investigate the potential for a dose response we chose a priori to assess the total number of weeks that training was undertaken, and the number of HIIT minutes per week that participants performed, with neither having any statistically significant relationship with CRF. Given that we have already demonstrated that the effect size due to HIIT is partially dependent upon the control group used as a comparator, we chose to run a posteriori meta-regression of just those studies using MICE as a control and just those studies using no-exercise as a control. However, these additional analyses were also non-significant indicating that no relationship between the effect of HIIT and the duration or number of HIIT minutes per week in the program. In the present analysis, duration ranged between 4 and 15 weeks. It may be that the adaptations in response to HIIT training are initially rapid but slower thereafter. The relatively small range of study duration limits the interpretation of the available literature. Furthermore, additional longer studies comparing MICE to HIIT are warranted, as given the present data, it is plausible that neither MICE nor HIIT is superior if compared over a long enough training duration. A further confounding factor is that all studies which examined longer durations (≥8 weeks) were also studies which used participants with low CRF at baseline, while those utilising shorter durations of training (2–7 weeks), were almost exclusively adolescents with high levels of CRF at baseline.

In contrast to the limited data on training duration, there was a much broader range of HIIT min week^−1^ included, ranging from 1.5–44 min week^−1^. However, despite the greater range there remained no evidence of a dose response. This may be due in part to the heterogeneity in training protocols. As may be anticipated, those studies with the highest amount of HIIT min week^−1^ tended to have lower HIIT intensities ranging between 82–95% $\dot{V}O_{2\max}$ whereas those with fewer min week^−1^ used maximal or supra maximal ‘all out’ sprint efforts. HIIT has been reported to produce an increase in CRF that is rapid and disproportionately large relative to the training volume [48,50]. Given such a non-linear response to HIIT training, it is perhaps not surprising that there was no evidence of a dose response in relation to the number of HIIT min week^−1^. From the studies included in the present meta-analysis, it is clear that protocols with a wide variation in HIIT min week^−1^ result in broadly equivalent changes in CRF. This analysis therefor supports the notion that both low and high volume school based HIIT appear to be equally effective.

The lack of relationship between CRF and either HIIT duration, HIIT program length or HIIT min week^−1^, has important implications for implementing school based HIIT. Given the lack of a dose response between 4 and 15 weeks programs, it suggests that school-based HIIT programs could be effective if delivered in 4 week blocks interspersed throughout the academic year. Furthermore, PE pedagogy is rightly concerned with much more than simply maintaining students CRF, meaning teachers may be cautious about devoting large amount of class time to CRF training. However, HIIT protocols have been delivered in as little as 6 min, and given the present findings, this indicates that such sessions are equally effective as longer duration protocols. It may be feasible for these short protocols to be effectively incorporated into current PE lessons [51,52], with minimal disruption to the taught curriculum, particularly if they are only required to be performed for 4 weeks at a time.

## 5. Strengths and Limitations of This Review and Meta-Analysis

The present meta-analysis has several strengths. The inclusion of surrogate measures of CRF means we have doubled the number of studies that could be included in the overall analysis. This also meant that it was possible to undertake more meaningful moderator and meta-regression analysis.

The review is not without limitations, however. While we were thorough in our search protocol, it is possible that studies were missed, in particular studies that were published in other languages. Further, there are some technical considerations in the current meta-analysis. Although in general this study found reporting standards where adequate, there were exceptions. One study [38] did not report the number of sessions per week, or the number of weeks of training, meaning it was not included in the meta-regression. In addition, there were two studies [22,35] that did not report HR, again making comparisons with previous research difficult. While not all investigators will prescribe training intensities based on HR, in such cases we support other recent calls [53] for studies of HIIT to at least include the actual HR achieved during training.

## 6. Conclusions

This systematic review and meta-analysis found HIIT has a statistically significant moderate effect on improving CRF in adolescents when compared to MICE and no exercise control, independent of baseline CRF or body weight. Furthermore, the similarities in findings between direct and predicted measures of CRF suggest large scale field-based studies are both sustainable and valid. Low volume HIIT, interspersed through the school year, alongside standard PE classes could be a time effective method of improving CRF in adolescents without significantly impeding regular PE pedagogy.

## Figures and Tables

**Table 1:** Risk of bias and Quality Appraisal assessment.

Study	A	B	C	D	E	F	G	H	Risk of Bias Total	Quality Appraisal
Baquet et al., (2001) [22]	x	✓	NA	x	✓	✓	✓	x	4	4
Boddy et al., (2010) [34]	x	✓	✓	✓	✓	✓	x	x	3	5
Boer et al., (2014) [35]	x	✓	NA	✓	✓	✓	✓	x	3	5
Buchan et al., (2011) [24]	x	✓	x	✓	✓	✓	✓	✓	2	6
Buchan et al., (2012) [25]	x	✓	x	✓	✓	✓	✓	✓	2	6
Buchan et al., (2013) [23]	x	✓	x	✓	✓	✓	✓	✓	2	6
Coute de Araujo et al., (2012) [36]	✓	✓	x	x	✓	✓	✓	x	3	5
Impellizzeri (2006) [37]	✓	✓	NA	✓	✓	✓	✓	✓	1	7
Koubaa et al., (2013) [38]	x	✓	✓	x	✓	✓	x	x	4	4
Martin et al., (2015) [27]	x	✓	x	✓	✓	✓	✓	✓	2	6
Martin-Smith et al., (2018) [28]	x	✓	x	✓	✓	✓	✓	✓	2	6
Murphy et al., (2015) [39]	x	✓	✓	NA	✓	✓	x	✓	3	5
Racil et al., (2013) [40]	✓	✓	NA	✓	✓	✓	✓	✓	1	7
Racil et al., (2016) [41]	✓	✓	NA	✓	✓	✓	✓	✓	1	7
Sandbakk (2013) [42]	x	✓	NA	✓	✓	✓	x	x	4	4
Sperlich (2011) [43]	x	✓	NA	✓	✓	✓	✓	✓	2	6
Starkoff et al., (2015) [44]	✓	✓	x	✓	✓	✓	x	x	3	5
Tjonna et al., (2009) [45]	✓	✓	NA	x	✓	✓	✓	x	3	5

Note: A = participants were randomly allocated to groups. B = the groups were similar at baseline. C = Blinding of assessor taking primary outcome. D = adequately powered groups. E = Included a non-exercising control or moderate intensity exercise control group. F = High intensity interval training (HIIT) level of intensity included. G = HIIT total time included. H = group × time interaction and effect size.

**Table 2:** Description of high intensity interval training (HIIT)/sprint interval training studies (SIT) in adolescents.

Author (Year)	Study Design (CT = Control Trial; RCT = Randomised control trial	Subjects Numbers (n)/Intervention Duration (weeks)	Weight Status (H = Healthy; O = Overweight; OB = Obese)	Mode	Percentile of CRF	Assessment of CRF (Direct = D; Indirect = I)	Protocol	HIT/SIT Sessions Week^−1^	Duration of HIT/SIT Intervals	Total Intervention Duration Including Rest	Number of HIT/SIT Intervals Per Session	Duration of Rest Intervals	Intensity of HIT/SIT Intervals
Baquet et al., (2001) [22]	CT	551 school adolescents Males (M) and Females (F) (12–15 years) HI = 503 (12.7 ± 1.1 years) CON = 48 (13 ± 1 years) 10 weeks	H	Running sprints	25th Percentile	I	HI= 3 × (10 s/10 s) @ 100–120% MAS) 3 min recovery C = 3 h of PE per week	1	10 s	60 min	3	3–5 min	100–120% MAS
Boddy et al., (2010) [34]	RCT	16 F (11.8 ± 0.3 years) INT = 8 CON = 8 5 weeks	H	Dance class	75th percentile	I	INT= 6 × 30 s of high intensity activities @ >80 mean % HRmax with 45 s recovery CON = no information provided	4	30 s	3 h	6	45 s	>80% HRmax
Boer et al., (2014) [35]	RCT	46 M & F adolescents (17 ± 3) SIT = 17 CAT = 15 CON = 14 15 weeks	H	Cycling	15th percentile	I	SIT = 10 × 15 s @ 110% VT with 45 s rest CAT 30 min CAT @ 100% VT CON = normal routine	2	15 s	5 h	10	45 s	110% VT
Buchan et al., (2011) [24]	CT	47 M & F adolescent scholars (16.4 ± 0.7 years) HIIT = 17 (16.7 ± 0.1 years) MOD= 16 (16.2 ± 0.1years) CONT= 24 (16.3 ± 0.5 years) 7 weeks	H	Running sprints	85th percentile	I	HIIT= 4–6 × 30/30 s running sprints at maximal effort ET= 20 min continuous running at 70% VO_2max_ CONT = normal daily routine	3	30 s	63	4 to 6	30 s	86.7% HRmax
Buchan et al., (2012) [25]	CT	41 M & F adolescent scholars (15–17 years) HIIT = 17 CONT = 24 7 weeks	H	Running sprints	85th percentile	I	HIIT = 4–6 × (30/30s) running sprints at maximal effort CONT = normal daily routine	3	30 s	63 min	4 to 6	30 s	86.8% HRmax
Buchan et al., (2013) [23]	CT	89 M & F Adolescent scholars HIIT = 42 (16.8 ± 0.5 years) CONT = 47 (16.6 ± 0.6 years) 7 weeks	H	running sprints	85th percentile	I	HIIT= 4–6 × (30/30 s) running sprints at maximal effort CONT = normal daily routine	3	30 s	63 min	4 to 6	30 s	86.7% HRmax
Coute de Araujo et al., (2012) [36]	RCT	39 M & F Obese children (8–12 years) HIIT = 20 (10.7 ± 0.7years) ET = 19 (10.4 ± 0.9 years) 12 weeks	OB	Treadmill sprints	10th percentile	I	INT = 4 × 60 s at 100% MAV 3 min at 50% of MAV (12 weeks) ET = 30–60 min of continuous running at 80% HRmax	2	60 s	60 min	3 to 6	3 min	100% MAS
Impellizzeri et al., (2006) [37]	CT	29 M & F Adolescents STG= 14 GTG= 15 Age: 17.8 ± 0.6years 8 weeks	H	Running sprints	95th percentile	D	STG = 4 × 4 min @ 90–95% HRmax GTC = normal training	2	4	128 min	4	3 min	95% HRmax
Koubaa et al., (2013) [38]	CT	29 M & F obese adolescents (13 ± 0.8 years) HIIT = 14 C = 15 12 weeks	OB	Running sprints	25th percentile	D	HIIT = 2 min work @80–100% VO_2max_ (reps not stated) C = continuous running (30 min at 60–70% VO_2max_)	3	2 min	-	-	60 s	100% VO2max
Martin et al., (2015) [27]	CT	49 M & F adolescent scholars SIT = 26 (16.8 ± 0.3 years) SPE =23 (17.0 ± 0.2 years) 7 weeks	H	Running sprints	85th percentile	I	SIT= 4–6 × (30/30 s) @ 86.5% HRmax) SPE= standard 3 h of PE per week	3	30 s	63 min	4 to 6	30 s	86.5% HRmax
Martin-Smith et al., (2018) [28]	CT	56 M & F adolescents INT = 24 (17 ± 0.3 years) CON = 32 (16.8 ± 0.5 years 4 weeks	H	Running sprints	85th percentile	I	INT= 5–6 × (30/30s) @ 92.2% HRmax CON= standard 3 h of PE per week	3	30 s	66 min	5–6	30 s	92.2% HRmax
Murphy et al., (2015) [39]	CT	13 M & F adolescents (14.4 years) HIIE = 7 (13.7 ± 2.0 years) AE = 6 (14.3 ± 2.0 years) 12 weeks	O	HIIE = Cycling SAE-continuous aerobic exercise	25th percentile	D	HIIE = 10 × 1 min @ 80–90% HRmax interspersed with 2 min @ 60% HRmax AE-continuous aerobic exercise	3	60 s	90 min	10	2 min	80–90% HRmax
Racil et al., (2013) [40]	RCT	34 obese F adolescents (15.9 ± 0.3 years) HIIT = 11 MIIT = 11 CG = 12 12 weeks	OB	Running sprints	25th percentile	I	HIIT = 2 × (6–8 × 30s/30s) @ 100–110% MAS MIIT = 2 × (6–8 × 30/30s) @ 70–80% MAS CG = normal daily activities	3	30 s	up to 4 h 24 min	6 to 8	30 s and 4 min	100% MAS
Racil et al., (2016) [41]	RCT	47 F (14.2 ± 1.2 years) HIIT = 17 MIIT = 16 CON = 14 12 weeks	OB	Running sprints	25th percentile	I	HIIT = 15 s/15 s @100% MAS/50% MAS MIIT = 15 s/15 s @800% MAS/50% MAS CON = no exercise	3	15 s	8 h	8 to 16	15 s	100% MAS
Sandbakk et al., (2011) [42]	CT	15 M & F adolescents CG = 8 IG = 7 Age: 17.4 ± 0.5 years 8 weeks	H	Cross country skiing	95th percentile	I	INT1 = 1.5–3 h @60–74% HRmax INT2 = 1–2 h continuous work @78–84% HRmax INT3 5–10 min interval at 85–92% HRmax	-	-	-	-	-	92% HRmax
Sperlich et al., (2011) [43]	CT	19 M & F adolescents HIIT = 9 HVT = 10 age:13.5 ± 0.4 years 5 weeks	H	Running sprints	95th percentile	D	HIIT = Variation of intervals at 90–95% HRmax HVT = various fartlek sessions at 50–70% HRmax lasting 45–60 min	3 to 4	30 s–4 min	166 min	4 to 12	30 s–4 min	95% HRmax
Starkoff et al., (2014) [44]	RCT	27 M and F adolescents (14.7 ± 1.5 years) HIIE = 14 (14.9 ± 1.6 years) MOD = 13 (14.5 ± 1.4 years) 6 weeks	OB	Cycling	25th percentile	D	HIIE = 10 × 2 min @95–100% APMHR interspersed with 1 min @55% APMHR MOD = 65–75% APMHR	3	2 min	5 h	10	1 min	95–100% APMHR
Tjonna et al., (2009) [45]	RCT	54 overweight/obese adolescents (14 ± 0.3years) AIT = 28 MTG = 26 12 weeks	O/OB	Treadmill running sprints	10th percentile	D	AIT = 4 × 4 min @ 90–95% HRmax with 3 min recovery @ 70% HRmax MTG = Activity sessions 3 times in 12 months and educational conversation groups	2	4 min	6 h 20 min	4	3 min	95% HRmax

NOTE: HI = High intensity. C = Control. INT = Intervention. CG = Control Group. CON = Control. CONT = Control. SIT = Sprint interval Training. IG = Interval Group. CAT = Continuous Aerobic Training. ET = Endurance Training. HI = High Intensity. STG = Sprint Training Group. GTG = Generic Training Group. SPE = Standard Physical Education. PE = Physical Education. HIIE = High Intensity Interval Exercise. MTG = Moderate Training Group. AIT = Aerobic Interval Training. AE = Aerobic Exercise. MIIT = Moderate Intensity Interval Training. MOD = Moderate Intensity. MAV = Maximal Aerobic Velocity. VT = Velocity Threshold. APMHER = Age Predicted Maximal Heart Rate. HVT = High Volume Training. AIT = Aerobic Interval Training. MAS= Maximal Aerobic Speed. MTG = Multidisciplinary Training Group.

**Table 3:** Cardiorespiratory fitness (CRF) PRE and POST Values in HIIT and CON groups.

Author (Year)	CRF HIIT(PRE) mL kg^−1^ min^−1^	CRF HIIT (POST) mL kg^−1^ min^−1^	CRF CON (PRE) mL kg^−1^ min^−1^	CRF CON (POST) mL kg^−1^ min^−1^	Improvement in CRF in HIIT Group (%)	Effect Size (*g*)	*p* Value
Baquet et al., (2001) [22]	37.7 ± 2.1	40.02 ± 2.7	38.34 ± 3.2	38.49±2.3	3.9	0.61	<0.001
Boddy et al., (2010) [34]	41.26 ± 4.67	42.59 ± 7.51	43.61 ± 9.01	45.71 ± 7.09	3.1	0.42	>0.05
Boer et al., (2014) [35]	31.5 ± 5.2	31.4 ± 4.8	28.7 ± 5.7	27.4 ± 4.6	0.3	0.84	<0.01
Buchan et al., (2011) [24]	47.1 ± 6.4	52.6 ± 6.76	49.9 ± 7.1	48.8 ± 7. 6	7.60	0.53	<0.001
Buchan et al., (2012) [25]	47.1 ± 6.4	52.6 ± 6.7	49.9 ± 7.1	48.8 ± 7.64.2	7.70	0.53	<0.001
Buchan et al., (2013) [23]	46.28 ± 6.9	53.1 ± 7.2	47.72 ± 7.2	43.67 ± 6.2	6	0.78	<0.001
Coute de Araujo et al., (2012) [36]	26.5 ± 3.9	30.1 ± 4.2	26.9 ± 3.6	31.1 ± 4.2	13.40	0.25	0.004
Impellizzeri (2006) [37]	57.7 ± 7.1	61.4 ± 4.6	55.6 ± 3.4	59.7 ± 4.1	7	0.48	> 0.05
Koubaa et al., (2013) [38]	38.7 ± 1.2	42.9 ± 1.7	37.5 ± 1.6	39.2 ± 3.2	9.80	1.43	<0.001
Martin et al., (2015) [27]	48.28 ± 6.84	51.81 ± 6.37	50.46 ± 5.96	46.77 ± 5.68	6.8	0.95	<0.05
Martin-Smith et al., (2018) [28]	47.13 ± 6.31	49.13 ± 6.22	46.10 ± 7.32	42.88 ± 7.14	4	0.93	<0.05
Murphy et al., (2015) [39]	29.1 ± 3.5	32.7 ± 4.0	26.8 ± 4.9	30.2 ± 2.6	11	0.95	> 0.05
Racil et al., (2013) [40]	29.8 ± 2.7	30.5 ± 2.9	30.5 ± 2.5	31.1 ± 2.7	2.2	0.2	<0.05
Racil et al., (2016) [41]	36.9 ± 1.8	39.7 ± 1.8	38.1 ± 1.5	38.6 ± 1.4	12.60	1.25	<0.05
Sandbakk (2013) [42]	67.5 ± 6.5	70.2 ± 6.8	69.3 ± 7.2	70.3 ± 7.3	3.8	0.01	> 0.05
Sperlich (2011) [43]	55.1 ± 4.9	58.9 ± 4.7	55.3 ± 4.3	56.43 ± 3.7	7	0.59	<0.001
Starkoff et al., (2015) [44]	20.0 ± 5.7	22.7 ± 6.5	19.5 ± 6.6	19.6 ± 7.6	11.9	0.4	<0.05
Tjonna et al., (2009) [45]	32.3 ± 5.8	35.3 ± 0.8	32.3 ± 4.8	32.3 ± 0.8	8.5	0.68	<0.001

## References

[1] Guthold R., Stevens G.A., Riley L.M., Bull F.C. (2019). Global trends in insufficient physical activity among adolescents: A pooled analysis of 298 population-based surveys with 1.6 million participants. Lancet Child Adolesc. Health.

[2] World Health Organisation (2010). Global Recommendation on Physical Activity for Health.

[3] Biddle S.J., Gorely T., Marshall S.J., Cameron N. (2009). The prevalence of sedentary behavior and physical activity in leisure time: A study of Scottish adolescents using ecological momentary assessment. Prev. Med..

[4] Dumith S.C., Gigante D.P., Domingues M.R., Hallal P.C., Menezes A.M., Kohl H.W. (2012). A longitudinal evaluation of physical activity in Brazilian adolescents: Tracking, change and predictors. Pediatric Exerc. Sci..

[5] Telama R., Yang X. (2000). Decline of physical activity from youth to young adulthood in Finland. Med. Sci. Sports Exerc..

[6] Trost S.G., Pate R.R., Sallis J.F., Freedson P.S., Taylor W.C., Dowda M., Sirard J. (2002). Age and gender differences in objectively measured physical activity in youth. Med. Sci. Sports Exerc..

[7] Fairclough S.J., Ridgers N.D., Welk G. (2012). Correlates of children’s moderate and vigorous physical activity during weekdays and weekends. J. Phys. Act. Health.

[8] Boyle S.E., Jones G.L., Walters S.J. (2008). Physical activity among adolescents and barriers to delivering physical education in Cornwall and Lancashire, UK: A qualitative study of heads of PE and heads of schools. BMC Public Health.

[9] Love R., Adams J., van Sluijs E.M.F. (2019). Are school-based physical activity interventions effective and equitable? A meta-analysis of cluster randomized controlled trials with accelerometer-assessed activity. Obes. Rev..

[10] Mura G., Rocha N.B., Helmich I., Budde H., Machado S., Wegner M., Nardi A.E., Arias-Carrion O., Vellante M., Baum A. (2015). Physical activity interventions in schools for improving lifestyle in European countries. Clin. Pract. Epidemiol. Ment. Health CP EMH.

[11] Ortega F.B., Ruiz J.R., Castillo M.J., Sjostrom M. (2008). Physical fitness in childhood and adolescence: A powerful marker of health. Int. J. Obes..

[12] Matton L., Thomis M., Wijndaele K., Duvigneaud N., Beunen G., Claessens A.L., Vanreusel B., Philippaerts R., Lefevre J. (2006). Tracking of physical fitness and physical activity from youth to adulthood in females. Med. Sci. Sports Exerc..

[13] Adab P., Pallan M.J., Lancashire E.R., Hemming K., Frew E., Barrett T., Bhopal R., Cade J.E., Canaway A., Clarke J.L. (2018). Effectiveness of a childhood obesity prevention programme delivered through schools, targeting 6 and 7 year olds: Cluster randomised controlled trial (WAVES study). Br. Med. J..

[14] Anderson E.L., Howe L.D., Kipping R.R., Campbell R., Jago R., Noble S.M., Wells S., Chittleborough C., Peters T.J., Lawlor D.A. (2016). Long-term effects of the Active for Life Year 5 (AFLY5) school-based cluster-randomised controlled trial. BMJ Open.

[15] Metcalf B., Henley W., Wilkin T. (2012). Effectiveness of intervention on physical activity of children: Systematic review and meta-analysis of controlled trials with objectively measured outcomes (EarlyBird 54). BMJ.

[16] Burgomaster K.A., Heigenhauser G.J., Gibala M.J. (2006). Effect of short-term sprint interval training on human skeletal muscle carbohydrate metabolism during exercise and time-trial performance. J. Appl. Physiol..

[17] Burgomaster K.A., Howarth K.R., Phillips S.M., Rakobowchuk M., Macdonald M.J., McGee S.L., Gibala M.J. (2008). Similar metabolic adaptations during exercise after low volume sprint interval and traditional endurance training in humans. J. Physiol..

[18] Burgomaster K.A., Hughes S.C., Heigenhauser G.J., Bradwell S.N., Gibala M.J. (2005). Six sessions of sprint interval training increases muscle oxidative potential and cycle endurance capacity in humans. J. Appl. Physiol..

[19] Gibala M.J., Little J.P., Macdonald M.J., Hawley J.A. (2012). Physiological adaptations to low-volume, high-intensity interval training in health and disease. J. Physiol..

[20] Whyte L.J., Gill J.M., Cathcart A.J. (2010). Effect of 2 weeks of sprint interval training on health-related outcomes in sedentary overweight/obese men. Metab. Clin. Exp..

[21] Gibala M.J., Little J.P., van Essen M., Wilkin G.P., Burgomaster K.A., Safdar A., Raha S., Tarnopolsky M.A. (2006). Short-term sprint interval versus traditional endurance training: Similar initial adaptations in human skeletal muscle and exercise performance. J. Physiol..

[22] Baquet G., Berthoin S., Gerbeaux M., Van Praagh E. (2001). High-intensity aerobic training during a 10 week one-hour physical education cycle: Effects on physical fitness of adolescents aged 11 to 16. Int. J. Sports Med..

[23] Buchan D.S., Ollis S., Young J.D., Cooper S.M., Shield J.P., Baker J.S. (2013). High intensity interval running enhances measures of physical fitness but not metabolic measures of cardiovascular disease risk in healthy adolescents. Bmc Public Health.

[24] Buchan D.S., Ollis S., Young J.D., Thomas N.E., Malina R.M., Baker J.S. (2011). The effects of time and intensity of exercise on novel and establihed markers of CVD in adolescent youth. Am. J. Hum. Biol..

[25] Buchan D.S., Young J.D., Simpson A.D., Thomas N.E., Cooper S.M., Baker J.S. (2012). The effects of a novel high intensity exercise intervention on established markers of cardiovascular disease and health in Scottish adolescent youth. J. Public Health Res..

[26] Delgado-Floody P., Latorre-Roman P., Jerez-Mayorga D., Caamano-Navarrete F., Garcia-Pinillos F. (2019). Feasibility of incorporating high-intensity interval training into physical education programs to improve body composition and cardiorespiratory capacity of overweight and obese children: A systematic review. J. Exerc. Sci. Fit..

[27] Martin R., Buchan D.S., Baker J.S., Young J., Sculthorpe N., Grace F.M. (2015). Sprint interval training (SIT) is an effective method to maintain cardiorespiratory fitness (CRF) and glucose homeostasis in Scottish adolescents. Biol. Sport.

[28] Martin-Smith R., Buchan D.S., Baker J.S., Macdonald M.J., Sculthorpe N.F., Easton C., Knox A., Grace F.M. (2019). Sprint Interval Training and the School Curriculum: Benefits Upon Cardiorespiratory Fitness, Physical Activity Profiles, and Cardiometabolic Risk Profiles of Healthy Adolescents. Pediatric Exerc. Sci..

[29] Costigan S.A., Eather N., Plotnikoff R.C., Taaffe D.R., Lubans D.R. (2015). High-intensity interval training for improving health-related fitness in adolescents: A systematic review and meta-analysis. Br. J. Sports Med..

[30] Eddolls W.T.B., McNarry M.A., Stratton G., Winn C.O.N., Mackintosh K.A. (2017). High-Intensity Interval Training Interventions in Children and Adolescents: A Systematic Review. Sports Med..

[31] Logan G.R.M., Harris N., Duncan S., Schofield G. (2014). A Review of Adolescent High-Intensity Interval Training. Sports Med..

[32] Moher D., Liberati A., Tetzlaff J., Altman D.G. (2009). Preferred reporting items for systematic reviews and meta-analyses: The PRISMA statement. Br. Med. J..

[33] Higgins J.P., Altman D.G., Gotzsche P.C., Juni P., Moher D., Oxman A.D., Savovic J., Schulz K.F., Weeks L., Sterne J.A. (2011). The Cochrane Collaboration’s tool for assessing risk of bias in randomised trials. BMJ.

[34] Boddy L.M., Stratton G., Hackett A.F., George K.P. (2010). The effectiveness if a “short, sharp, shock” high intensity exercise intervention in 11-and 12 year old Liverpool school girls. Arch. Exerc. Health Dis..

[35] Boer P.H., Meeus M., Terblanche E., Rombaut L., Wandele I.D., Hermans L., Gysel T., Ruige J., Calders P. (2014). The influence of sprint interval training on body composition, physical and metabolic fitness in adolescents and young adults with intellectual disability: A randomized controlled trial. Clin. Rehabil..

[36] Coute de Araujo A.C., Roschel H., Picanco A.R., do Prado D.M., Villares S.M., de Sa Pinto A.L., Gualano B. (2012). Similar health benefits of endurance and high-intensity interval training in obese children. PLoS ONE.

[37] Impellizzeri F.M., Marcora S.M., Castagna C., Reilly T., Sassi A., Iaia F.M., Rampinini E. (2006). Physiological and performance effects of generic versus specific aerobic training in soccer players. Int. J. Sports Med..

[38] Koubaa A., Trabelsi H., Masmoudi L., Elloumi M., Sahnoun Z., Zeghal K.M., HaKim A. (2013). The effects of intermittent and continuous training on body composition, cardiorespiratory fitness and lipid profile in obese adolescents. IOSR J. Pharm..

[39] Murphy A., Kist C., Gier A.J., Edwards N.M., Gao Z., Siegel R.M. (2015). The feasibility of high-intensity interval exercise in obese adolescents. Clin. Pediatrics.

[40] Racil G., Ben Ounis O., Hammouda O., Kallel A., Zouhal H., Chamari K., Amri M. (2013). Effects of high vs. moderate exercise intensity during interval training on lipids and adiponectin levels in obese young females. Eur. J. Appl. Physiol..

[41] Racil G., Coquart J.B., Elmontassar W., Haddad M., Goebel R., Chaouachi A., Amri M., Chamari K. (2016). Greater effects of high- compared with moderate-intensity interval training on cardio-metabolic variables, blood leptin concentration and ratings of perceived exertion in obese adolescent females. Biol. Sport.

[42] Sandbakk O., Sandbakk S.B., Ettema G., Welde B. (2013). Effects of intensity and duration in aerobic high-intensity interval training in highly trained junior cross-country skiers. J. Strength Cond. Res..

[43] Sperlich B., De Marees M., Koehler K., Linville J., Holmberg H.C., Mester J. (2011). Effects of 5 weeks of high-intensity interval training vs. volume training in 14-year-old soccer players. J. Strength Cond. Res..

[44] Starkoff B.E., Eneli I.U., Bonny A.E., Hoffman R.P., Devor S.T. (2014). Estimated Areobic Capacity Changes in Adolescents with Obesity Following High Intensity Interveal Exercise. Int. J. Kinesiol. Sports Sci..

[45] Tjonna A.E., Stolen T.O., Bye A., Volden M., Slordahl S.A., Odegard R., Skogvoll E., Wisloff U. (2009). Aerobic interval training reduces cardiovascular risk factors more than a multitreatment approach in overweight adolescents. Clin. Sci..

[46] Egger M., Davey Smith G., Schneider M., Minder C. (1997). Bias in meta-analysis detected by a simple, graphical test. BMJ.

[47] Cohen J. (1988). Statistical Power Analysis for the Behavioural Sciences.

[48] Weston K.S., Wisloff U., Coombes J.S. (2014). High-intensity interval training in patients with lifestyle-induced cardiometabolic disease: A systematic review and meta-analysis. Br. J. Sports Med..

[49] Tomkinson G.R., Olds T.S. (2007). Secular changes in pediatric aerobic fitness test performance: The global picture. Med. Sport Sci..

[50] Bacon A.P., Carter R.E., Ogle E.A., Joyner M.J. (2013). VO2max trainability and high intensity interval training in humans: A meta-analysis. PLoS ONE.

[51] Costigan S.A., Ridgers N.D., Eather N., Plotnikoff R.C., Harris N., Lubans D.R. (2018). Exploring the impact of high intensity interval training on adolescents’ objectively measured physical activity: Findings from a randomized controlled trial. J. Sports Sci..

[52] Leahy A.A., Eather N., Smith J.J., Hillman C., Morgan P.J., Nilsson M., Lonsdale C., Plotnikoff R.C., Noetel M., Holliday E. (2019). School-based physical activity intervention for older adolescents: Rationale and study protocol for the Burn 2 Learn cluster randomised controlled trial. BMJ Open.

[53] Taylor K.L., Weston M., Batterham A.M. (2015). Evaluating intervention fidelity: An example from a high-intensity interval training study. PLoS ONE.

